# Supplementary material for: Phytochemical Analysis, α-Glucosidase and α-Amylase Inhibitory Activities and Acute Toxicity Studies of Extracts from Pomegranate (Punica granatum) Bark, a Valuable Agro-Industrial By-Product
Source: Foods. 2022 May 6;11(9):1353. doi: 10.3390/foods11091353 (PMC9103815; doi:10.3390/foods11091353)
Supplement: Supplementary file 1 [file foods-11-01353-s001.zip › foods-1687480-supplementary.pdf]

Supplementary Materials

Phytochemical Analysis, α-Glucosidase and α-Amylase Inhibitory Activities and Acute Toxicity Studies of Extracts from Pomegranate *(Punica granatum)* Bark, a Valuable Agro-Industrial By-Product

Nassima Laaraj [1], Mohamed Bouhrim [2], Loubna Kharchoufa [2], Salima Tiji [1], Hasna Bendaha[1], Mohamed Addi [3], Samantha Drouet [4], Christophe Hano [4,*], Jose Manuel Lorenzo [5,6], Mohamed Bnouham [2] and Mostafa Mimouni [1,*]

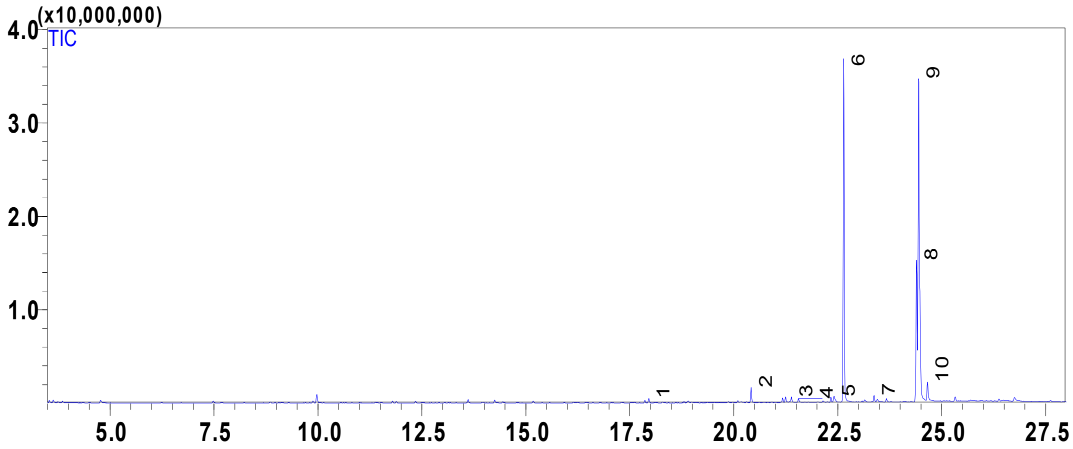

Figure S1: Chromatogram of the defatted hexane fraction (HEE) of pomegranate bark by GC-MS.

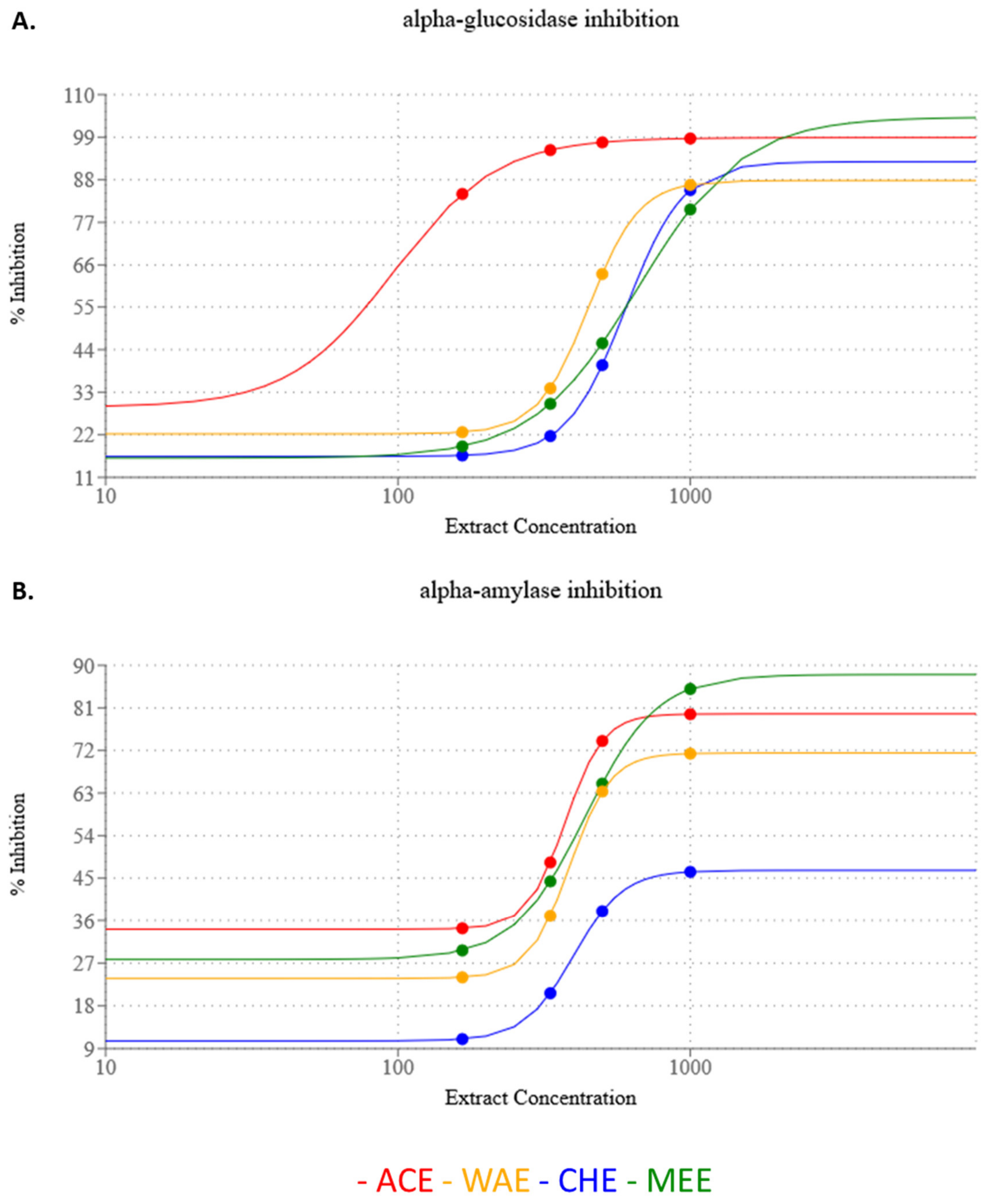

Figure S2: IC_{50} curves for inhibition of (**A**) intestinal α-amylase and (**B**) pancreatic α-amylase by pomegranate *(Punica granatum)* acetone (ACE), water (WAE), chloroform (CHE) and methanol (MEE) bark extracts. Each experiment was done in triplicate.

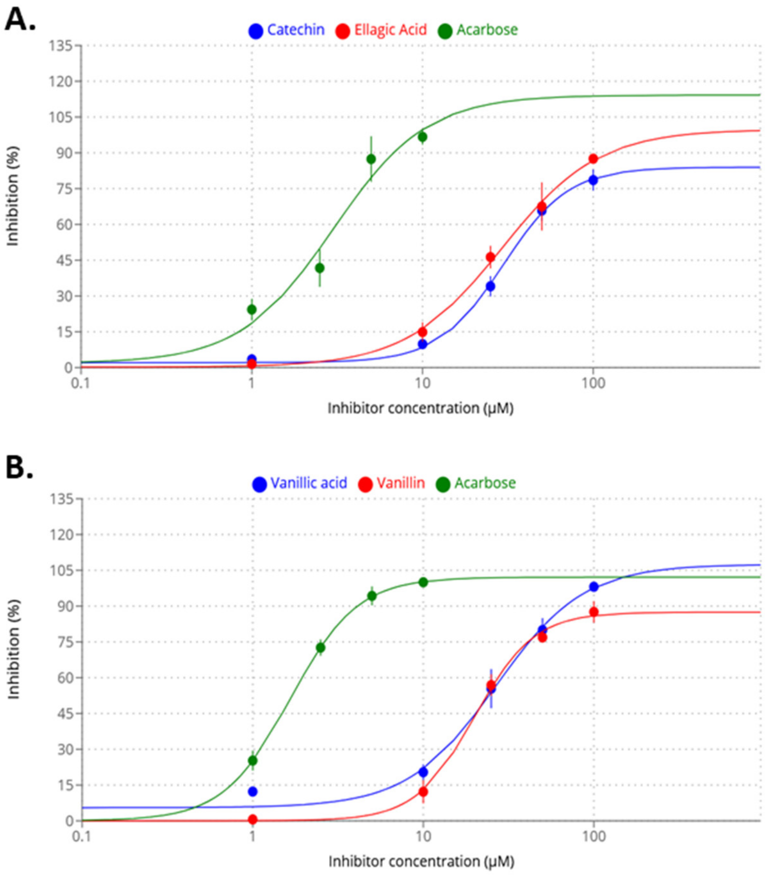

Figure S3: IC_{50} curves (without background correction (subtract smallest response) to show error bars) for inhibition of (**A**) intestinal α-amylase by acarbose, (-)-catechin and ellagic acid, and of (**B**) pancreatic α-amylase by acarbose, vanillic acid and vanillin. Each experiment was done in triplicate.

Table S1: Chemical composition percentage (%Area) identified in the defatted hexane fraction (HEE) by GC-MS.

Pic Number	Name	Retention time (mn) (RT)	%Area
1	Lauric acid	17.992	0.32
2	Myristic acid	20.542	1.40
3	Tetracosane	21.308	0.63
4	6-Octadecenoic acid,	21.467	0.79
5	7-Hexadecenoic acid	22.517	1.08
6	Palmitic acid,	24.333	31.72
7	10-Methylicosane	23.417	0.67
8	Linoleic acid,	24.417	14.37
9	Oleic acid	27.958	46.97
10	Stearic acid	24.842	2.05
